# Analysing electrocardiographic traits and predicting cardiac risk in UK biobank

**DOI:** 10.1177/20480040211023664

**Published:** 2021-06-12

**Authors:** Julia Ramírez, Stefan van Duijvenboden, William J Young, Michele Orini, Aled R Jones, Pier D Lambiase, Patricia B Munroe, Andrew Tinker

**Affiliations:** 1 Clinical Pharmacology, William Harvey Research Institute, Barts and The London School of Medicine and Dentistry, Queen Mary University of London, London, UK; 2 Institute of Cardiovascular Science, University College London, London, UK; 3 Barts Heart Centre, St Bartholomew’s Hospital, London, UK; 4 NIHR Barts Cardiovascular Biomedical Research Unit, Barts and The London School of Medicine and Dentistry, Queen Mary University of London, London, UK

**Keywords:** Arrhythmias, clinical electrophysiology, drugs, genomics, ion channels, membrane transport

## Abstract

The electrocardiogram (ECG) is a commonly used clinical tool that reflects cardiac excitability and disease. Many parameters are can be measured and with the improvement of methodology can now be quantified in an automated fashion, with accuracy and at scale. Furthermore, these measurements can be heritable and thus genome wide association studies inform the underpinning biological mechanisms. In this review we describe how we have used the resources in UK Biobank to undertake such work. In particular, we focus on a substudy uniquely describing the response to exercise performed at scale with accompanying genetic information.

## Introduction

Sudden cardiac death is an important health problem accounting for 1,00,000 deaths per annum in the UK.^1^ The commonest cause is ischaemic heart disease, but, in patients under the age of thirty-five years, cardiomyopathies and channelopathies predominate. Implantable cardioverter defibrillators have revolutionised management in many disease settings but, despite much research, many are implanted in patients who do not suffer an event.^2^ Furthermore, idiopathic cardiac arrest occurs in 30–40%. Thus, there is important clinical need for better risk stratification. The surface electrocardiogram (ECG) is a non-invasive and important clinical technique used to assess cardiac excitability. The nuanced interpretation of abnormal patterns in the ECG may provide the necessary prognostic tool that could be performed at scale.

UK Biobank is a prospective large-scale health study in which half a million individuals aged 40–69 years old were recruited in 2006–2010. These participants gave detailed health information and underwent a series of investigations, including providing blood and urine samples, cognitive testing, etc. and expanded with a series of sub-studies, including on-going online questionnaires, linkage with health records and activity monitors.^3^ Specifically, an exercise ECG was performed in ∼90,000 individuals as part of the original study and, more recently, a standard 12 lead ECG as part of an on-going imaging sub-study. This is complemented by genetic information, initially using Affymetrix UKBiLEVE Axiom array and later the UK Biobank Axiom array on all participants & extended with exome sequencing with data expected to be available in the full UK Biobank cohort in 2021 and whole genome sequencing to follow. Disease coding is implemented using the WHO International Classification of Diseases and Related Health Outcomes, Tenth Revision (ICD-10), which includes an extensive hierarchical tree-structured dictionary for cardiovascular diseases, including arrhythmia and sudden cardiac death. In this review, we describe our analyses on the ECG datasets in UK Biobank. We focus, firstly, on the genetics underpinning standard ECG measurements at rest, during and after exercise using genome wide association studies (GWASs) to elucidate loci and associated genes. The ability to interrogate the behaviour of the cardiac electrical system during exercise and recovery at scale with corresponding genetic information is a unique resource. Secondly, we ask what prognostic information for cardiovascular disease and, more specifically, sudden cardiac death, is contained in the ECG signal and its underlying genetics.

## What we measure and how

**Figure 1.**
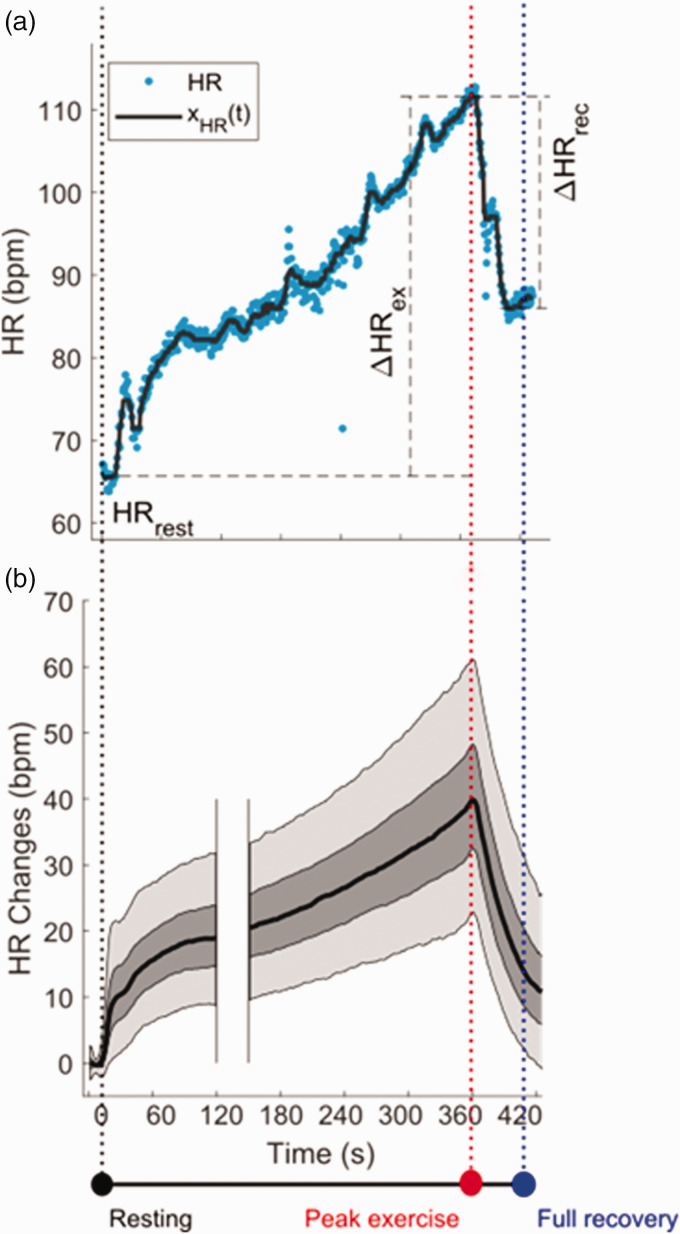
Heart rate profile and indices. (a) Heart rate (HR) profile and HR markers during the exercise stress test. The heart rate profile, x_HR_(t) (solid dark line) is a function of time obtained by filtering the instantaneous HR (dots). HR_rest_: Mean x_HR_(t) over 15 sec resting; HR_rec_: minimum x_HR_(t) during recovery; ΔHR_ex_ = HR at peak exercise − HR_rest_: HR dynamics during exercise; ΔHR_rec_ = HR at peak exercise − HR at full recovery: HR dynamics during recovery. (b) Distribution of the heart rate profile across all participants. Black solid line, dark and light shadowed areas represent median, 25th–75th percentiles and 5th–95th percentile intervals, respectively. Adapted from Orini et al.^4^

**Figure 2.**
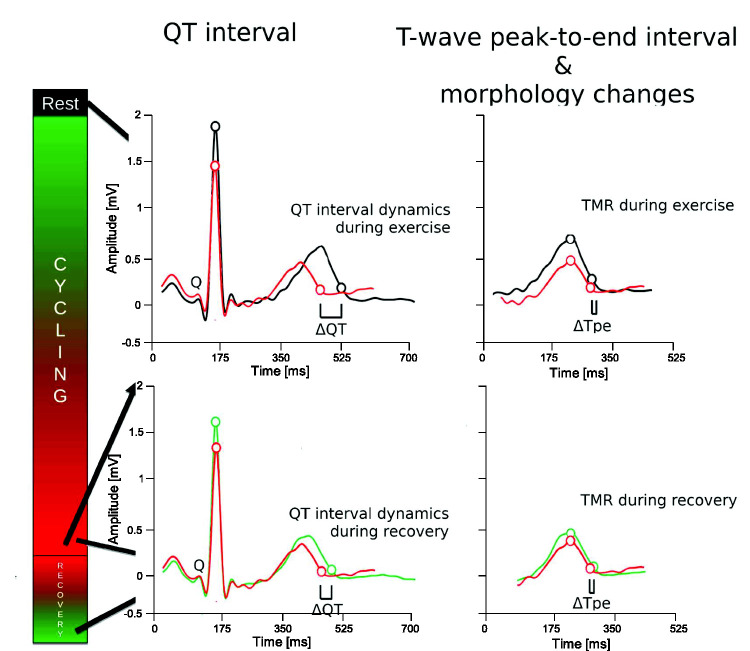
Schematic illustration of markers of ventricular repolarisation dynamics during exercise and recovery. Three averaged heartbeats were derived at rest (black), peak exercise (red), and full recovery (green), respectively. QT and T-peak-to-end (Tpe) intervals were measured for each of the three heartbeats. The QT and Tpe dynamics during exercise were calculated as the difference between the QT and Tpe intervals, respectively, at rest and peak exercise, divided by the corresponding change in RR interval (not shown here). Dynamics during recovery were calculated in a similar fashion but using the intervals measured at recovery instead of rest. Morphological changes between the T waves at rest, peak exercise and recovery were quantified by the T-wave morphology restitution (TMR) index.

Figures 1 and 2 illustrate the measurements we made and these are discussed in more detail below.

### Exercise stress test

A total of 95,216 individuals were invited for an exercise test using a four-lead electrocardiograph during cycle ergometry on a stationary bike. The test followed a standardized protocol during which the workload was gradually increased up to either 30 or 50% of the predicted maximum workload, and a 1-minute recovery period without pedalling. Throughout the protocol, workload and heart rate were recorded. Raw digital ECG recordings enabled detailed analysis of exercise related biomarkers. We focused on subjects without a history of known cardiovascular disease as this affects heart rate., Automatic quantification of the ECG indices was performed as follows:^4–7^

1. ***Computation of signal-averaged ECG waveforms***: Signal averaging is a standard and effective technique to reduce noise and artefacts from ECG recordings. ECG waveforms of successive heartbeats within short intervals of interest (e.g. 10–20 s at rest, peak exercise or recovery) are carefully aligned and the signal-averaged waveform is computed across heartbeats showing very similar waveforms, i.e. showing high correlation (e.g. r > 0.90) to the median waveform template. This ensures that ectopic beats, artefacts and noisy segments do not affect the signal-averaged waveforms.^8–10^
2. ***Automatic annotation of ECG markers :*** ECG waves are annotated using algorithms developed within our team^8–10^ and PR, QRS, QT, RT, T wave peak-end (Tpe) are measured. Algorithms for the measurement of QRS duration, QT and RT are freely available and have been shown to be accurate when compared to expert annotation.^11^
3. ***Computation of advanced repolarization markers:*** We have implemented a methodology to quantify T-wave morphological changes due to heart rate changes^12^ and we have demonstrated that it is a strong predictive risk factor for sudden cardiac death in patients with heart failure.^13^ This methodology uses non-linear warping to provide a measurement of morphological difference between two T-waves.
4. **Quantification of response to heart rate:** Response to exercise was calculated by dividing the difference between each corresponding ECG measurement at rest and at peak exercise by the change in the RR interval (inverse of heart rate) during exercise. Similarly, response to recovery was calculated by dividing the difference between each corresponding ECG measurement at peak exercise and at recovery by the change in the RR interval during recovery.

### Standard 12-lead ECG

We pre-processed and signal averaged heartbeats in the 10 second recordings as in the exercise stress test ECGs. The onset, peak, and end timings of the waveforms were located using the same bespoke software as in previous studies.^8,9^

### Reproducibility of measurements with time

Interestingly, within the exercise cohort, a small group of approximately ∼1,000 individuals were invited for a repeat exercise stress test ∼3 years later. Reproducibility of heart date dynamics markers is important to support their use in genetic studies or as a clinical biomarker. We therefore examined the Intra-individual correlation between heart rate profile during the first and the second assessment.^14^ High intra-individual reproducibility was found and the intra-individual correlation between the profiles was also higher than inter-individual correlation (0.92 ± 0.08 vs 0.87 ± 0.11, p < 0.01).^14^ This suggests that heart rate dynamics markers are indeed subject-specific which may add valuable clinical information.

## Heart rate and response to exercise

Reduced heart rate dynamics during exercise and recovery from exercise (heart rate recovery) are strongly associated with both all-cause and cardiovascular mortality.^15^ The inability to either increase heart rate during exercise or slow during recovery is thought to reflect an imbalance of the autonomic tone. In Figure 1 we show the mean response and illustrate the degree of variability within the study population. We and another group have investigated the genetic basis of heart rate changes during exercise and recovery independently in UK Biobank.^4,16^ Both markers were found to have a heritable component with an estimated heritability up to 17%. In our study, 30 independent loci were discovered, 8 of which were common to both markers.^4^ Bioinformatic analyses implicated several candidate genes important in neural development and modulation of adrenergic activity by the autonomic nervous system. For example, one of the prioritised genes for HR dynamics during exercise is *BTB* (broad complex, tramtrack and bric à brac) Domain Containing 9 (*BTBD9*). *BTB* proteins play an important role in synaptic plasticity and neurotransmission and *BTBD9* is among pathways related to the regulation of the circadian rhythm, known to be involved in cardiac parasympathetic modulation. Another candidate gene is the Ca^2+^-dependent activator protein for secretion 1 (*CAPS1*) gene at the *FUT5* locus. *CAPS1* is present in neurones and endocrine cells, and is involved in mediating exocytosis from large dense-core vesicles, which in the adrenal medulla affect catecholamine release. It is, therefore, plausible that the association between *CAPS1* and heart rate response to recovery is mediated by the sympathetic nervous system. The other study had similar findings and also implicated the autonomic nervous control.

## QT interval and exercise

Abnormalities in cardiac repolarisation have long been recognised as a risk factor for sudden cardiac death. Both significant lengthening and shortening of the QT interval have been associated with increased risk of sudden cardiac death with hereditary Mendelian syndromes and in populations with and without known cardiovascular disease.^1^ Evidence from linkage analyses show there is a clear heritable component accounting for ∼35% of the variability of the QT interval in the general population.^17^ *NOS1AP*, the first locus identified to be associated with QT using a GWAS approach and subsequently consistently replicated across cohorts of different ancestries, had not previously been recognised to be involved in cardiac repolarisation.^18^ The coded protein (CAPON) mediates interactions with neuronal nitric oxide synthase (nNOS), but the underlying biological mechanisms involved in its relationship with QT remains unclear.^19^ As *NOS1AP* variants are non-coding, they may influence transcriptional effects, and functional evaluation suggests over-expression causes attenuation of L-type calcium current.^19^ Despite some uncertainty of the biological mechanisms, variants in *NOS1A*P are estimated to account for a significant proportion of the variability of resting QT (∼1.5%) and are associated with an increased risk of drug-induced QT prolongation, ventricular arrhythmia and SCD in white adults.^20^ Subsequent QT GWAS and exome-wide analyses of larger sample sizes, including from consortia, have highlighted the roles of cardiac ion channels, calcium signalling pathways and myocardial structural proteins in modulating the resting QT interval.^21^ To date, loci identified through published GWAS collectively explain ∼9% of resting QT-interval variation. A significant proportion of the heritability is unexplained and the potential remains to identify new biological processes with larger samples using improved imputation methods.

Abnormal heart rate dependency of the QT interval (measured by the slope of the QT/RR profile) is observed in patients at risk for cardiac death and arrhythmic events but its study in populations of generally healthy individuals had not been explored until recently. Using the UK Biobank exercise test cohort, we recently published a study of this marker.^7^ Figure 2 illustrates how the measurements were made. QT dynamics was not a significant independent predictor of cardiovascular risk, suggesting its prognostic importance may relate to individuals with an underlying arrhythmogenic substrate, such as those with existing ischaemic heart disease. However, its genetic study did reveal insight into biological processes involved in QT dynamics. The heritability of QT dynamics at exercise and recovery is less compared with resting QT (10.7% and 5.4% respectively), but despite this, 5 novel loci were identified not previously reported for resting QT, with candidate genes including *KIAA1755* which is highly expressed in brain and nerve tissue, supporting a potential role in autonomic regulation. Of interest, this study also identified overlap of loci with resting QT, such as those encoding ion channels or channel-interacting proteins. Additionally, QT dynamics and response to exercise differs between females and males. Sex-stratified analyses identified a significant locus (*FOXN3*) for QT dynamics on exercise in males only though the biology underlying this is unclear.

## T-wave

Abnormal T-wave morphologies on the ECG are a risk marker for ventricular arrhythmic mortality and all-cause mortality, independent of age, sex, comorbidities, QRS duration and corrected QT interval, not only in healthy subjects,^22^ but also in individuals with acquired QT prolongation^23^ and cardiac disease.^24^ Although the general view is that the T-wave reflects spatial dispersion of ventricular repolarization; the exact nature of this is disputed.^9^ One pre-eminent suggestion is that it reveals differences in transmural repolarization, but this is largely based on the *ex-vivo* ventricular wedge preparation and has not been reproduced in the intact heart.^25^

The T-peak-to-T-end (Tpe) interval measures the distance between the peak and the end of the T-wave, and is heritable^6^ (Figure 2). In the largest study to date, we identified 28 loci and 4 male-specific loci contributing to the Tpe interval. From these, 12 were also associated with resting QT, 2 with resting heart rate, 5 with QRS complex and 3 with the PR interval, indicating shared genetic architecture among ECG traits.^6^ However, 10 loci were specific to the Tpe interval (i.e. not previously reported for any other ECG trait), and 8 had plausible candidate genes (*PPP1R3B/MFHAS1*, *PYGB*, *KCNJ4*, *GATA4*, *RUFY1*, *SERTAD2*, *GPR1/ZDBF2* and *HEY2)*. Bioinformatics analyses on all identified loci confirmed that cellular processes that control ventricular repolarization predominantly drive the main biological mechanism underlying the Tpe interval.^6^ In particular, the lead SNV at *KCNJ2* demonstrated the strongest association with the Tpe interval and one of the largest effect sizes for this trait (1.3 milliseconds). *KCNJ2*, *KCNH2* and *RNF207* are all associated with the Tpe interval, as well as with the QT interval.^21^ An additional biological mechanism confirmed by bioinformatics analyses underlying the Tpe interval is the gene ontology term “cardiac conduction and contraction”. Several candidate genes, such as *PYGB*, *GATA4*, and *HEY2*, as well as previously reported *SCN5A-SCN10A*, *CAMK2D* and *KCND3* are involved.

Our recent work studied the genetic contribution to the Tpe response to exercise and to recovery from exercise.^6^ We found the heritability of these two traits was low (2.2% for Tpe response to exercise and 2.4% for Tpe response to recovery), suggesting there is a significant genetic contribution to resting Tpe, but its response to heart rate changes is mainly influenced by environmental factors. Still, three loci were identified for Tpe response to exercise and three loci for Tpe response to recovery with little overlap with other ECG traits. One of the candidate genes for Tpe response to exercise is *ETS2*, which is part of a genetic network governing cardiopoiesis.^26^

Another measure is the restitution of the T-wave morphology, i.e. how it changes with heart rate, and is a strong predictive risk factor for sudden cardiac death in patients with heart failure^27^ (Figure 2). The T-wave morphology restitution index (TMR) quantifies the rate of variation of the overall T-wave morphology with heart rate^5^ and hence captures more information than the Tpe response to heart rate. We performed a GWAS on TMR during exercise and during recovery and again found low heritability estimations (3.5% and 4.9%, respectively),^5^ confirming largely environmental drivers. Despite low heritability, 12 loci associated with both traits were identified, 4 of which were common to both markers.^5^ Again, genetic variations at 4 of the 8 loci identified for TMR during exercise overlapped with long-QT syndrome and QT in the general population: *KCNH2*, *KCNJ2*, *SCN5A*, and *KCNQ1*, all known regulators of action potential repolarisation.^1^ For TMR during recovery, a variant at *KLF12* had previously been reported to be associated with the QT interval, the ST-T segment, and QRS duration.

## Biological insights

In general, the SNPs linked to ECG traits are tag SNPs, and not necessarily the causal variant. In contrast, they identify a genomic region in which one or more genes may be present.^28^ The experience of analysing the “FTO locus” in obesity indicates that the causative gene can be at a considerable genomic distance.^29^ However, even considering this, the results of our studies confirm existing biology and suggest new pathways for analysis.

**Figure 3.**
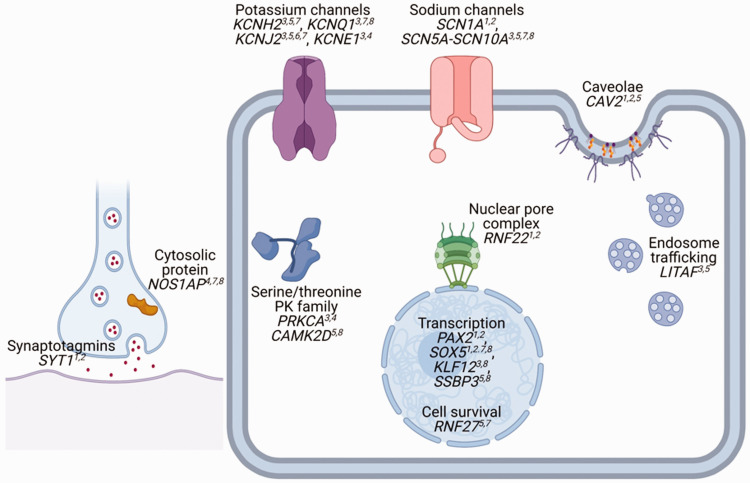
Overlap of candidate genes for dynamic traits and Tpeak-Tend interval. The figure shows overlap of candidate genes for each ECG measure. 1. Heart rate response to exercise, 2. Heart rate response to recovery, 3. QT dynamics on exercise, 4. QT dynamics on recovery, 5. Resting Tpeak-Tend interval, 6. Tpeak-Tend dynamics on exercise, 7. T-wave morphology restitution index (TMR) on exercise, 8. TMR on recovery. PK; Protein kinase. Graphic created using BioRender.com.

We have mentioned a considerable number of individual loci and genes above associated with specific traits but it is worth making some general comments. There is some overlap of the loci underpinning the various ECG traits as detailed in Table 1 and Figure 3 shows how these might integrate functionally. The QT interval and T wave reflect various aspects of ventricular repolarisation. A number of potassium currents govern this process, including IKs constituted of proteins encoded by *KCNQ1* and *KCNE1* genes, IKr from *KCNH2* and IK1 from *KCNJ2*. During exercise, rate accumulation and adrenergic activation of IKs oppose the increase in the L-type calcium current to reduce action potential duration at higher heart rates. Thus, it is not surprising that *KCNQ1* and *KCNE1* are implicated in the response of the QT interval and the T wave to exercise.^5–7^ The T wave is thought to reflect spatial variations in ventricular repolarisation, though the exact nature is disputed, with advocates for transmural, apex to base and left versus right ventricular differences.^30^ In our GWAS of Tpe the strongest signal implicated *KCNJ2*.^6^ This suggests differences in expression of IK1 in different regions of the ventricles may be important in influencing the T wave. A detailed functional analysis of this locus may be revealing and whether regional expression lay behind it.

**Table 1.** Overlap of loci underlying dynamic ECG traits.

	HR response to exercise	HR response to recovery	QT dynamics exercise	QT dynamics recovery	Resting Tpeak-Tend interval	Tpeak-Tend dynamics exercise	TMR exercise	TMR recovery
*RNF22*	1	1						
*SCN1A*	1	1						
*SNCA1P*	1	1						
*CAV2*	1	1			1			
*PAX2*	1	1						
*SOX5*	1	1					1	1
*SYT1*	1	1						
*MCTP2*	1	1						
*SCN5A-SCN1A*			1		1		1	1
*SLC35F1*			1		1			
*KCNH2*			1		1		1	
*KCNQ1*			1				1	1
*KLF12*			1					1
*LITAF*			1		1			
*PRKCA*			1	1				
*KCNJ2*			1		1	1	1	
*KCNE1*			1	1				
*NOS1AP*				1			1	1
*RNF27*					1		1	
*SSBP3*					1			1
*CAMK2D*					1			1

However, for many genes and loci, the mechanism of any potential effect is obscure and there is the potential for the discovery of new biology. For example, we isolated striatin as a potential causative gene in determining the resting Tpe interval. Striatins are thought to be important scaffolding proteins and have multiple protein interaction domains including ones binding caveolins and calcium-calmodulin, a coiled-coil domain and a tryptophan-aspartate domain.^31^ Intriguingly, a deletion in the 3’ untranslated region of striatin has been found to be associated with canine cardiomyopathies.^32^ Furthermore, other SNPs in striatin are linked with QRS duration and PR interval as well as various structural neurological features (www.ebi.ac.uk/gwas). In muscle striatin interacts with sarcolemmal membrane associated protein which directs it to the sarcolemma, t-tubule and sarcoplasmic reticulum.^33^ Mutations in the latter have been associated with Brugada syndrome and overexpression downregulated cardiac sodium channel expression.^34^ Thus it is possible to build a plausible case for the importance of striatin in cardiac function but definitive experimentation is required. There are many other GWAS associations where a similar exercise is possible to varying degrees but it remains a problem of how to prioritise those for investigation.

## Risk prediction including genetic risk scores

**Figure 4.**
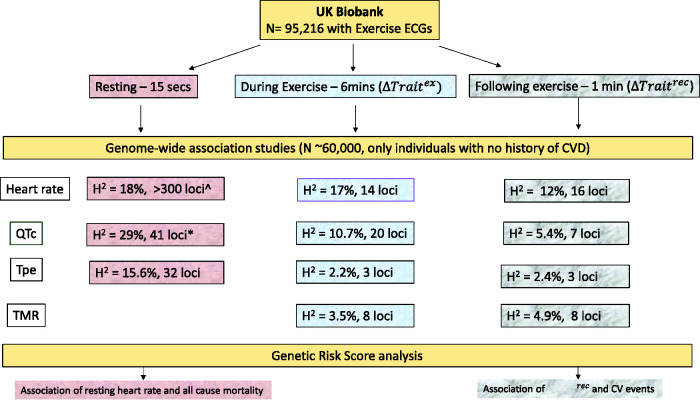
Overview of genetic analyses in the UKB exercise study. The number of loci are those which are genome-wide significant (P value). H2 = heritability; ^ indicates approx. number of resting heart rate loci (results from all UK Biobank sample^35^); * indicates number of loci discovered in the exercise study (unpublished); QTc = corrected QT interval, Tpe = T-waves peak and end, TMR = T-wave morphology restitution index, ΔTrait ex = difference in trait from resting and peak exercise; ΔTrait rec - difference in trait from peak exercise and 1 min following exercise; CV = cardiovascular.

With increasing numbers of loci being discovered for both resting and dynamic ECG traits in UK Biobank, these SNPs are being incorporated into genetic risk scores (GRSs) for assessing associations with disease outcomes and potential use for risk prediction. We summarise our studies and approach in Figure 4. Genetic variants discovered from GWASs individually are not informative for assessing risk; instead, a combined set of variants is required. To combine information across loci, GRSs or polygenic risk scores (PRSs) are created. There are several methods available to do this, which essentially summarise multiple genetic effects into a single score, usually this is the sum of the trait-associated genetic variants an individual carries weighted by estimated effect sizes of the variant.^36^ The application of ECG derived GRSs have yielded limited positive associations to date, with most of the studies utilising data from UK Biobank. There could be several reasons for this, firstly there is a true absence of association between current ECG derived GRSs and the tested cardiovascular outcomes. However, many of the tested GRSs explain a low percent of trait variance and there are relatively low numbers of individuals with clinical outcomes in UK Biobank currently, a study described as having a “healthy volunteer” selection bias.^37^

A review of results using scores from dynamic ECG traits indicates GRSs for heart rate response to exercise and recovery (using only genome-wide significant variants) demonstrates significant differences when comparing individuals in top and bottom quintiles for heart rate during exercise and recovery traits, the same GRSs however were not associated with cardiovascular mortality.^4^ This analysis should be regarded as exploratory as the same samples were used for creation of the GRSs and for testing. Furthermore, there was limited power as the percent variance explained for each trait was low (∼0.9%) and the number of individuals with the selected clinical outcomes was low (118 cases in the cohort). A nominally significant association was observed with a GRS for TMR response to recovery and cardiovascular events in individuals in the top 20% of the GRS compared to the bottom 20% (HR = 1.07, *P* = 6 × 10^–3^). A GRS was constructed including over 2,000 genetic variants in ∼60,000 individuals who participated in the exercise stress test with testing performed in remaining independent samples in UK Biobank (N ∼3,60,000). Using a different method for creation of a GRS with only genome-wide significant variants for QT response to exercise, gave no association with cardiovascular events.^7^ This analysis had a similar sample size as the TMR response to recovery GRS, indicating that a GRS for TMR response to recovery may be useful to explore further for cardiovascular risk prediction.

A review of results utilising resting ECG trait GRSs with disease outcomes indicates significant association of resting heart rate with all-cause mortality in UK Biobank (HR = 1.18, *P* = 3.22 x 10^–6^ with a weighted GRS.^38^ This GRS was calculated using variants with P < 10^–5^. A GRS for resting PR interval has been tested with selected cardiovascular traits, and several significant associations were found.^39^ The most significant associations were shorter PR interval and increased risk of atrial fibrillation and longer PR interval with increased risk of distal conduction disease. The GRS explained 62% of trait related variance, which is much higher than many of the GRSs for other ECG traits described therein, increasing power even though the numbers of events for many of the traits tested was relatively low (for example, 307 cases with atrioventricular preexcitation, *P* = 8.36 x 10^–4^). In UK Biobank with limited follow up data thus far many clinical outcomes of interest including sudden cardiac death and ventricular arrhythmias were relatively low when many of the ECG risk scores were tested.

Most GRSs across complex traits currently have relatively low “area under the curve” values but they are expected to improve with further genetic discoveries. In the short term, GRSs have demonstrated some clinical utility, importantly identifying individuals with a higher genetic risk for a trait. This success has been nicely demonstrated by studies of GRSs for coronary artery disease (CAD). Individuals with an intermediate CAD risk based on their GRS have been identified, with many individuals potentially benefiting from early targeted intervention with statins.^40^ The inclusion of GRSs with clinical, biochemical and lifestyle factors has been demonstrated to improve risk prediction, and identifies individuals who can be targeted for preventative interventions.^41^ Looking forward, the numbers of loci being discovered for ECG traits will increase, there will be opportunities for testing of current and new GRSs in other cohorts, and in patients with high risk which will provide knowledge of further disease associations. This new information can then be used to create new algorithms (using ECG GRSs, other cardiovascular trait GRSs, clinical and lifestyle factors) for improved risk prediction of cardiovascular traits.

## Conclusion

We have studied a range of ECG markers in UK Biobank datasets as reviewed above and summarised in Figure 4. One interesting feature is that the heritability of many ECG traits was reduced during exercise compared to rest, suggesting that environmental influences are much more important and suggesting exercise training may be able to modify them. Large population-based studies, such as the UK Biobank, provide opportunity for the study at scale of other and less understood ECG markers of cardiovascular risk, such as ventricular ectopy and its burden during exercise, and markers of global electrical heterogeneity.^42^ The latter has become of increasing interest with artificial intelligence approaches to predict arrhythmia from the standard 12 lead ECG.^43^ Therefore, there is potential for much more to be understood of the relationship between the ECG and cardiovascular risk going forward. The one limitation is that UK Biobank is a relatively healthy population and some of these markers may be much more predictive in a disease setting such as heart failure.
